# Minimally invasive single-session double-level rotational osteotomy of the forearm bones to correct fixed pronation deformity in congenital proximal radioulnar synostosis

**DOI:** 10.1007/s11832-016-0750-8

**Published:** 2016-06-16

**Authors:** Sherif N. G. Bishay

**Affiliations:** Department of Orthopaedics, National Institute of Neuromotor System, 51 Al-Madina Al-Menawara Street, Al-Mouhandeseen, Imbaba, Giza, 12411 Egypt

**Keywords:** Congenital radioulnar synostosis, Rotational forearm osteotomy

## Abstract

**Background:**

Congenital proximal radioulnar synostosis is the most common congenital disease of the elbow joints and forearms.

**Methods:**

This was a prospective study of 12 consecutive children (14 forearms) who presented to the National Institute of Neuromotor System in Egypt between September 2012 and September 2013 with severe congenital proximal radioulnar synostosis, having a mean pronation deformity of 70.7° (range 60°–85°), and who underwent operative correction by single-session double-level rotational osteotomy and percutaneous intramedullary K-wires of both the radius and ulna. Ten forearms were type III, and four were type II according to Cleary and Omer classification. The mean age at the time of surgery was 5 years and 2 months (range 4 years and 10 months to 6 years and 5 months). They were evaluated for functional results after rotational corrective osteotomy at a mean interval of 30.4 months (range 24–36 months) by physical examination and radiographs.

**Results:**

All children had a mean pronation deformity correction of 59.8° (range 30°–90°) reaching a final position of 20°–30° of pronation in the affected dominant extremities and 20° of supination in the affected non-dominant extremities after osteotomy. All children showed improvement in functional activities, with no loss of correction or non-union in any child, and no circulatory disturbances, neuropathies, or hypertrophic scars.

**Conclusion:**

Minimally invasive single-session double-level rotation osteotomy of the proximal ulna and distal radius with percutaneous intramedullary K-wire fixation is a safe, technically simple and efficient procedure which corrects pronation deformity.

## Introduction

Congenital proximal radioulnar synostosis, although a rare congenital disease, is the most common congenital disorder of the elbow joints and forearms [1]. It results in a fixed position of the forearm ranging from neutral rotation at the mid-prone position to severe fixed pronation deformity [2].

If the deformity is mild, little disability will be evident, as the ipsilateral shoulder and wrist can compensate effectively [3]. However, with significant pronation, daily activities such as eating, washing, dressing and accepting objects in the palm of the hand can be severely impaired [4].

The aim of the study was to evaluate the results of single-session double-level rotational osteotomy and intramedullary K-wires of both the bones distal to the site of the synostosis in order to bring the forearm into an optimal functional position for improving functional abilities.

## Patients and methods

This was a prospective study of 12 consecutive children (fourteen forearms) who presented to the National Institute of Neuromotor System in Egypt between September 2012 and September 2013 with severe congenital proximal radioulnar synostosis, having a mean pronation deformity of 70.7° (range 60°–85°), and who underwent operative correction of the resulting fixed pronation deformity by single-session double-level rotational osteotomy and intramedullary K-wires of both the radius and ulna. Ten forearms were type III, and four were type II according to Cleary and Omer classification. The institute provides health services to handicapped children throughout Egypt, which explains the relatively large number of cases collected from one center. The results in this group after at a mimimum follow-up of 2 years were reported in September 2015 using their medical records, and clinical and plain radiographic examinations.

The study included eight boys and four girls with a mean age at surgery of 5 years and 2 months (range 4 years and 10 months to 6 years and 5 months). The right forearm was involved in all 12 children and the left in two children. There was bilateral involvement in one boy and one girl. All children were right handed.

### Preoperative clinical examination

The mean preoperative range of motion of the elbow joint was from 3.1° extension (range 2°–4°) to 134.2° flexion (range 130°–140°). The mean preoperative pronation deformity was 70.7° (range 60°–85°). The pronation deformity was measured with the patient’s elbow held fixed to the side of the chest, the forearm at 90° and the angle between the longitudinal axis of the humerus and the line of the radial and ulnar styloid processes was measured with a goniometer, as described by Ogino and Hikino [3].

### Preoperative radiography

**Fig. 1:**
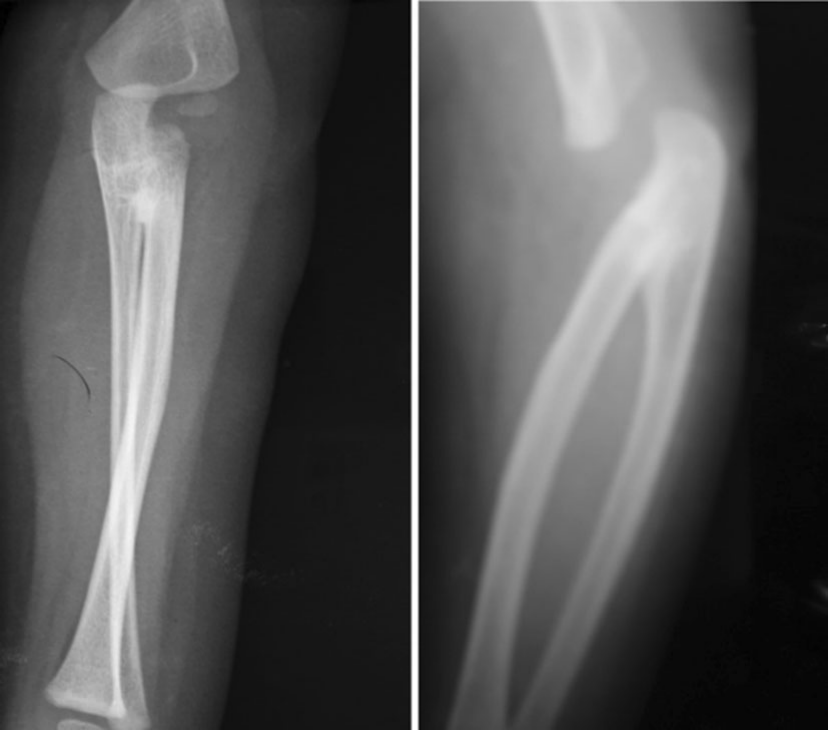
Preoperative anteroposterior (*left*) and lateral (*right*) views showing the bone synostosis and the radial head posterior dislocation (Cleary and Omer type III)

Standard anteroposterior and lateral radiographic views of the elbow and forearm were taken. Ten forearms (8 in boys and 2 in girls) were classified as type III according to Cleary and Omer [2] (Table 1) with visible osseous synostosis associated with posterior dislocation of a hypoplastic radial head (Fig. 1), while four forearms (2 in boys and 2 in girls) were type II, with visible osseous synostosis but without radial head dislocation.

**Table 1:** Cleary and Omer classification of congenital radioulnar synostosis [2]

Type	Criteria
Type I	There is a lack of involvement of the bone, and the radial head is located and normal
Type II	There is a visible osseous synostosis with a normal radius
Type III	There is an osseous synostosis with a hypoplastic and posteriorly dislocated radial head
Type IV	There is a short osseous synostosis with an anteriorly dislocated radial head

### Operative technique

Under general anesthesia with the patient supine, with a well-padded tourniquet, the operative steps were carried out in the following order:

1. *The ulnar approach* Under C-arm image guidance, a 2-mm K-wire was inserted percutaneously through the olecranon process into the medullary canal of the ulnar shaft and was advanced distally to stop just proximal to the proposed ulnar osteotomy site.The proximal ulna was approached through a very small longitudinal incision along its subcutaneous border. The ulnar osteotomy was marked distal to the site of the synostosis by multiple drill holes.
2. *The radial approach* Under C-arm image control, a 2-mm K-wire was inserted percutaneously through the distal radius into the medullary canal of the radial shaft and was advanced proximally to stop just distal to the proposed radial osteotomy site. The distal radius was approached through a very small longitudinal incision along the dorsolateral ridge of its distal third. The radial osteotomy was marked at the distal diaphyseal−metaphyseal junction by multiple drill holes.
3. *The osteotomy* The division of the radius first and then the ulna was completed using an electric saw or a very sharp osteotome.
4. *The forearm positioning* While keeping the arm position unchanged, the forearm was rotated to 20° pronation in the affected dominant extremities, or to 20° supination in the affected non-dominant extremities. The ulnar intramedullary K-wire was advanced distally to the distal third of the ulna until it came out through the ulnar styloid to be withdrawn distally percutaneously at the wrist so that its proximal end passed the olecranon process (being no more at the elbow) and the radial intramedullary wire proximally to the proximal third of the radius under the C-arm image control (Fig. 2).

**Fig. 2:**
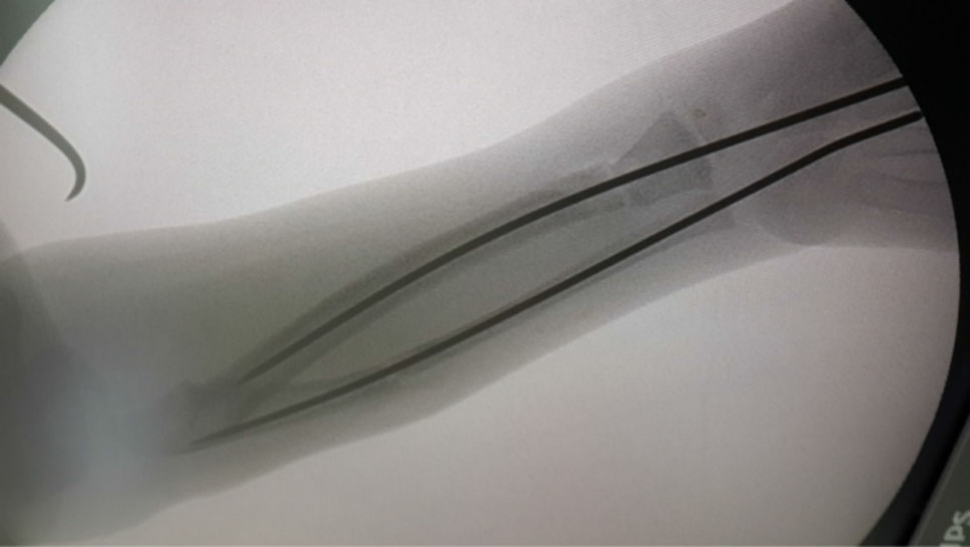
C-arm image control advancement of K-wires after the rotational osteotomy
5. *Hemostasis and wound closure* The tourniquet was deflated, and hemostasis was achieved. The two small wounds were closed with subcuticular sutures. A long above-elbow Plaster of Paris (POP) cast was placed over sterile dressings.

### After care

Strict observation for edema and peripheral circulation was started in the immediate postoperative period. Radiographs were performed every 3 weeks until complete consolidation of osteotomies. The POP cast was changed after 2 weeks to inspect the skin wound for healing. Cast and K-wires were removed at approximately 8 weeks, when bone consolidation was reached.

## Results

**Fig. 3:**
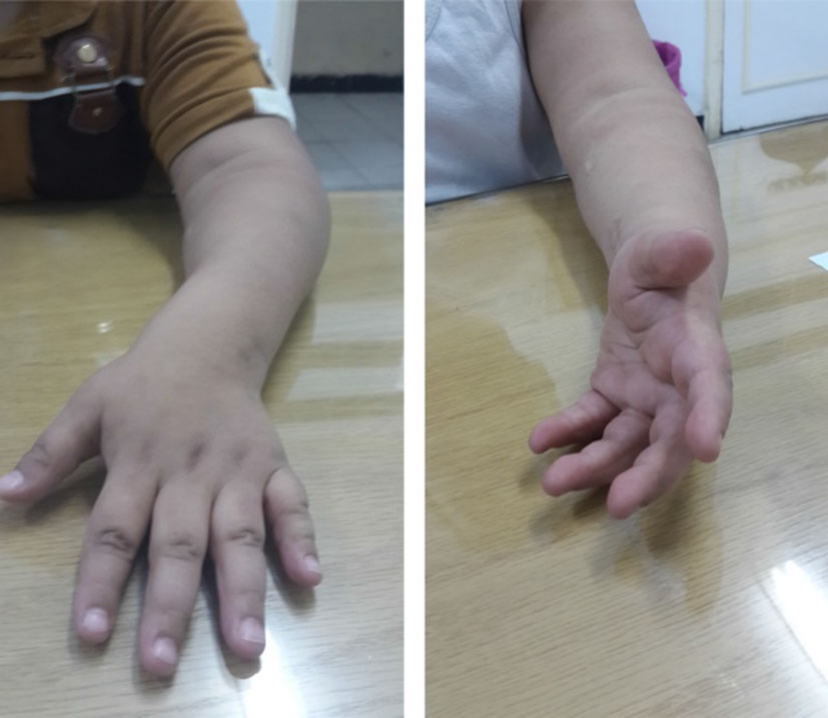
Preoperative fixed pronation (*left*) and postoperative midprone position (*right*)

**Fig. 4:**
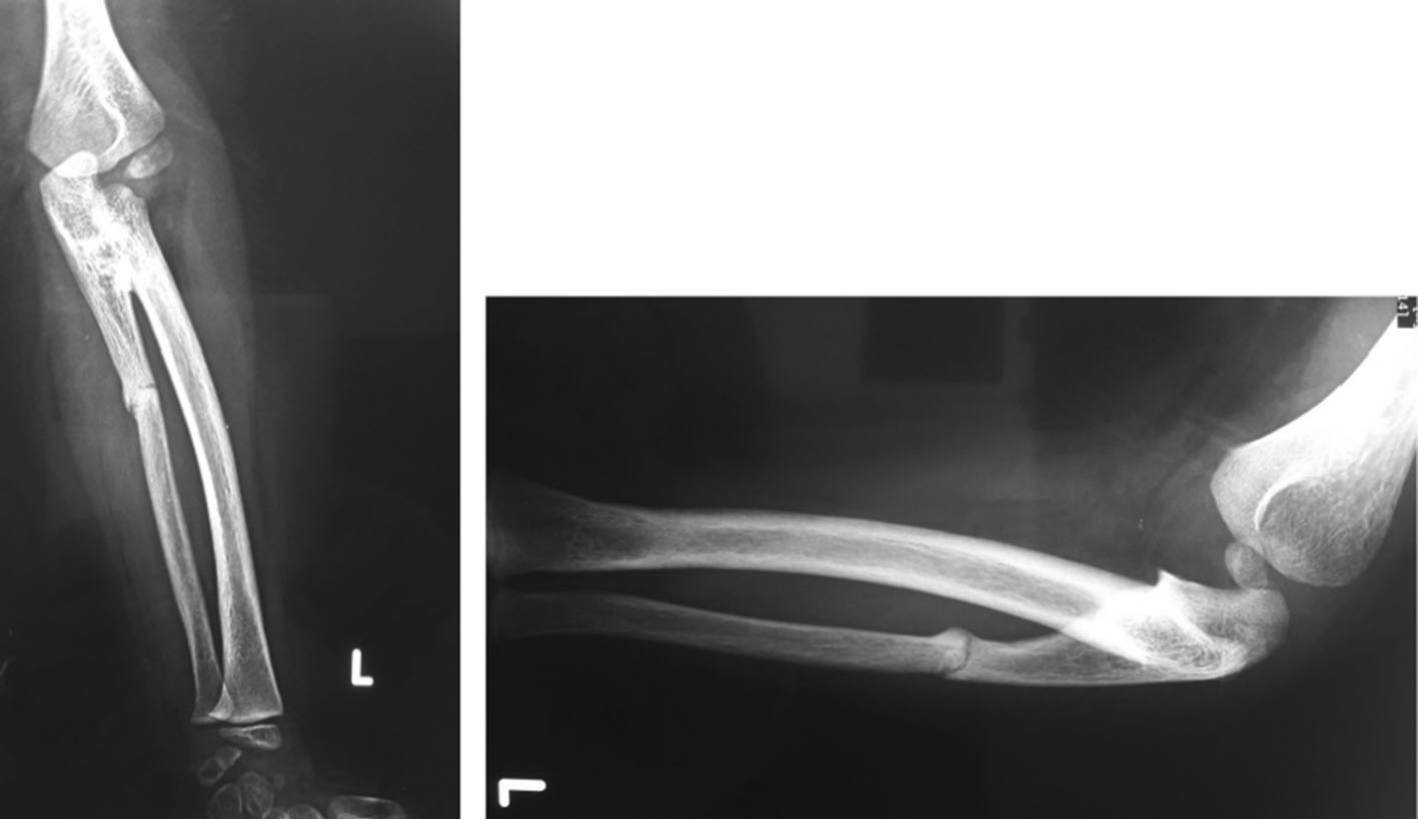
Postperative (8 weeks) anteroposterior (*left*) and lateral (*right*) views showing the rotation and union

The mean duration of follow-up was 30.4 months (range 24–36 months). Bone union was achieved in all patients, with a mean duration of 6.9 weeks (range 6–8 weeks). The mean time for complete removal of the cast was 6.5 weeks (range 6–8 weeks). The mean correction achieved after surgery was 59.8° (range 30°–90°) with a final position of 20°–30° of pronation in the affected dominant extremities and 20° of supination in the affected non-dominant extremities (Table 2). Elbow movements (extension and flexion) and wrist movements (dorsiflexion, palmarflexion, adduction, and abduction) were unaffected by the operation. At follow-up, there was no loss of correction (Fig. 3) or radiographic non-union (Fig. 4) in any child, and no circulatory disturbances, neuropathies, or hypertrophic scars on the forearm. All children showed marked functional improvement compared with the preoperative state, particularly in their daily activities such as eating, washing, dressing and accepting objects in the palm of the hand. All children and their families were satisfied with the results.

**Table 2:** Patient details

Patient no.	Age (years, months)	Gender	Side	Radiographic classification (Cleary and Omer)	Follow-up (months)	Preoperative fixed pronation deformity		Postoperative fixed pronation deformity	
						Right (°)	Left	Right (°)	Left
1	5, 2	M	Right	Type III	36	75	N	2	N
2	4, 11	M	Right	Type III	35	6	N	25	N
3	6, 5	M	Right	Type II	34	80	N	20	N
4	5, 4	M	Bilateral	Type III	33	65	70°	20	20° supination
5	4, 6	F	Bilateral	Type II	32	60	65°	20	20° supination
6	5, 7	M	Right	Type III	31	80	N	25	N
7	5, 3	M	Right	Type III	30	75	N	25	N
8	5, 1	M	Right	Type III	29	70	N	25	N
9	4, 10	F	Right	Type III	28	65	N	20	N
10	5, 0	M	Right	Type III	27	65	N	20	N
11	6, 2	M	Right	Type III	26	85	N	30	N
12	4, 11	F	Right	Type II	24	70	N	25	N

*M* male, *F* female, *N* normal

### Statistical results

Intra-observer and inter-observer variability were studied. The 14 forearms in the 12 children were examined and scored independently by four observers. On a separate occasion, two of the observers repeated the assessments of the same forearms in the absence of information from the initial observations. The overall intra-observer mean weighted kappa was *χ*_w_ = + 0.64 (range SE *χ* = 0.012–0.054) and the overall inter-observer mean weighted kappa was *χ*_w_ = + 0.54 (range SE *χ* = 0.009–0.041). The *p* value was <0.004.

## Discussion

Congenital proximal radioulnar synostosis is a rare congenital disease characterized by a fixed position of the forearm ranging from neutral rotation at the mid-prone position to fixed maximum pronation [2].

It is thought to be caused by a failure of prenatal longitudinal segmentation with persistence of the cartilaginous anlage between the radius and ulna during the seventh week of embryogenesis [5]. The resultant bridge may be fibrous or bony [6]. A genetic basis has been reported and attributed to the evidence of family history and the frequent association with other congenital anomalies and chromosomal abnormalities such as multiple X–Y syndromes [7]. However, in the current study, no case was associated with any other congenital anomaly.

The condition can be extremely disabling, especially in bilateral cases or in severe hyperpronation which occurs in 50–80 % of cases. Children who have a severe deformity have trouble bringing objects to the mouth or accepting objects into an open palm [4].

Old classification considered the synostosis as either type I with true bony fusion in which the radius and ulna are smoothly joined proximally for a variable distance, or type II in which there is congenital dislocation of the radial head with the synostosis just distal to the proximal radial epiphysis [6, 8, 9]. A more recent classification by Cleary and Omer [2] described four radiographic types as shown in Table 1. The current study showed that Cleary and Omer classification has poor clinical relevance. In fact, no differences were found in functional results of surgery when comparing the two treated types [10].

The indication for surgery depends on the severity of the deformity and the amount of disability. According to Farzan et al. [11], patients with congenital radioulnar synostosis who have no severe deformity and functional limitation need no surgical treatment. Simmons et al. [4] found that pronation of 60° was a definite indication for derotation osteotomy, while pronation of 15°–60° was a relative indication based on the needs of the individual. Ogino and Hikino [3] considered that the mean pronation of patients who complained of disability was 60° and of patients without complaints was 20°. Surgery is usually adjusted to individual needs. In the current study, all children had a significant disability with a mean pronation deformity of 70.7°.

The suitable age for surgical interference was believed by Griffet et al. [12] to be between 4 and 10 years, but Farzan et al. [11] recommended between the ages of 5 and 7 years. In the current study, the mean age was 5 years and 2 months, which is a relatively early age in order to have less neurovascular complications [13].

Various surgical modalities have been used to achieve rotation of the forearm [14]. Several authors reported separation of the synostosis and interposition of fascial or muscular flap, but recurrence of the ankylosis were noted [8, 10, 15, 16]. Hansen and Andersen [8] performed a partial resection of the left radius in a 16-year-old girl. Eighteen months postoperatively, osseous contact was noted in the follow-up plain radiography. Miura et al. [10] operated on eight upper extremities in seven patients. They placed the anconeus muscle between the separated radius and ulna, but the synostosis recurred in every patient. Kelikian and Doumanian [17] reported good results with use of a swivel prosthesis in patients who had post-traumatic proximal radioulnar synostosis; however, Tachdjian [18] noted disappointing results with the swivel prosthesis in patients who had a congenital synostosis, with recurrence of the ankylosis at the 18-month follow-up examination.

Rotational osteotomies to position the forearm in a more functional position are an alternative to separation of the synostosis. Three types of osteotomy procedures have been described to correct forearm rotation. The first type is osteotomy at the synostosis [19, 20], the second type is osteotomy at two sites in the diaphysis of the radius and the ulna [21–26], and the third type is osteotomy at one site in the distal diaphysis of the radius [27]. Rotational osteotomy at the synostosis is a technically complex surgical procedure over a narrow segment, and causes postoperative complications, including vascular compromise such as Volkmann’s compartmental ischemia, shortening and angulation of the forearm, and nerve palsy [3, 4, 26, 28]. In the double-level rotational osteotomy at two sites, the procedure is easier and there are fewer complications, although internal fixation is necessary, requiring a second surgery to remove the implant [27]. Green and Mital [14] suggested that in bilateral cases the best position was in 30°–45° of pronation in the dominant forearm and in 20°–35° of supination in the non-dominant forearm. In unilateral cases, the ideal position was 10°–20° of supination. Ogino and Hikino [3], Lin et al. [22], and Murase et al. [23] advocated 0°–20° of supination in the non-dominant forearm and 0°–20° of pronation in the dominant forearm. Ramachandran et al. [29] preferred a position of 10° supination in all cases as compensatory movements at the shoulder and wrist to allow the forearm to be located ideally for most daily activities. However, they found that hypermobility of the wrist was subjectively noted in all their patients.

Wael [24] performed two-stage double-level rotational osteotomy of both the radius and ulna without K-wire fixation, depending only on the POP cast for correction, and reported loss of correction in cases of cast loosening. Hung [25] performed single-stage double-level osteotomy with resection of a segment from both radius and ulna; a complex step with subsequent shortening of the forearm.

In the current study, all cases underwent double-level rotational osteotomy of the proximal ulna and the distal radius through very small and limited skin incisions, with minimal fixation by percutaneous intramedullary K-wires around which the corrective rotation took place. All cases also then underwent application of an above-elbow POP cast to maintain the corrected position, without further surgery for implant removal, as the K-wires were easily removed in the postoperative period by simple withdrawal through the skin. The results of the current study were satisfactory. The final position achieved after surgery was 20°–30° pronation in the affected dominant extremities and 20° of supination in the affected non-dominant extremities. In all cases, no patients reported disabilities in using the forearms and hands in eating, washing the face, and writing. All children and/or their parents were satisfied with the final position of their forearms. The technique was easy and safe, with the absence of postoperative complications and two small scars.

## Conclusion

Minimally invasive single-session double-level rotation osteotomy of the proximal ulna and distal radius with percutaneous intramedullary K-wire fixation is a safe, simple and effective procedure for the correction of fixed pronation in congenital proximal radioulnar synostosis to a position of good functional activity.
